# Role of Withaferin A and Its Derivatives in the Management of Alzheimer’s Disease: Recent Trends and Future Perspectives

**DOI:** 10.3390/molecules26123696

**Published:** 2021-06-17

**Authors:** Rajib Das, Abdur Rauf, Saima Akhter, Mohammad Nazmul Islam, Talha Bin Emran, Saikat Mitra, Ishaq N. Khan, Mohammad S. Mubarak

**Affiliations:** 1 Department of Pharmacy, Faculty of Pharmacy, University of Dhaka, Dhaka 1000, Bangladesh; rajibjony97@gmail.com (R.D.); saikatmitradu@gmail.com (S.M.); 2 Department of Chemistry, University of Swabi, Anbar 23561, Pakistan; mashaljcs@yahoo.com; 3 Department of Pharmacy, International Islamic University Chittagong, Chittagong 4318, Bangladesh; saima.iiuc16@gmail.com; 4 Department of Pharmacy, BGC Trust University Bangladesh, Chittagong 4381, Bangladesh; 5 Institute of Basic Medical Sciences, Khyber Medical University, Peshawar 25100, Pakistan; ishaqkhan.ibms@kmu.edu.pk; 6 Department of Chemistry, The University of Jordan, Amman 11942, Jordan

**Keywords:** withaferin A, Alzheimer’s disease, beta-amyloid, mevalonate, non-mevalonate pathway

## Abstract

Globally, Alzheimer’s disease (AD) is one of the most prevalent age-related neurodegenerative disorders associated with cognitive decline and memory deficits due to beta-amyloid deposition (Aβ) and tau protein hyperphosphorylation. To date, approximately 47 million people worldwide have AD. This figure will rise to an estimated 75.6 million by 2030 and 135.5 million by 2050. According to the literature, the efficacy of conventional medications for AD is statistically substantial, but clinical relevance is restricted to disease slowing rather than reversal. Withaferin A (WA) is a steroidal lactone glycowithanolides, a secondary metabolite with comprehensive biological effects. Biosynthetically, it is derived from *Withania somnifera* (Ashwagandha) and *Acnistus breviflorus* (Gallinero) through the mevalonate and non-mevalonate pathways. Mounting evidence shows that WA possesses inhibitory activities against developing a pathological marker of Alzheimer’s diseases. Several cellular and animal models’ particulates to AD have been conducted to assess the underlying protective effect of WA. In AD, the neuroprotective potential of WA is mediated by reduction of beta-amyloid plaque aggregation, tau protein accumulation, regulation of heat shock proteins, and inhibition of oxidative and inflammatory constituents. Despite the various preclinical studies on WA’s therapeutic potentiality, less is known regarding its definite efficacy in humans for AD. Accordingly, the present study focuses on the biosynthesis of WA, the epidemiology and pathophysiology of AD, and finally the therapeutic potential of WA for the treatment and prevention of AD, highlighting the research and augmentation of new therapeutic approaches. Further clinical trials are necessary for evaluating the safety profile and confirming WA’s neuroprotective potency against AD.

## 1. Introduction

Dementia is a progressive or chronic syndrome associated with reduced cognitive ability beyond the considerable consequence of normal aging. Approximately 50 brain diseases can cause dementia, of which Alzheimer’s disease (AD) is perhaps the most frequent among elderly patients and is likely to account for 50–70% of all cases of dementia [1]. AD is a chronic, progressive, irreversible, neurodegenerative disorder that is seen in age-dependent dementia. Early illness shows short-term memory loss, mood swings, inability to learn new information, difficulty in finding words, difficulty in solving problems, and disorientation in time and space [2,3,4,5,6]. Patients in severe stages suffer from serious memory loss, hallucinations, disorientation, and lack of self-sufficiency [5,7,8]. AD eventually leads to ample incapability and death of patients within 3 to 9 years after diagnosis [9]. To date, AD is the sixth leading cause of death [10,11]. Since 2014, approximately 36 million people worldwide have been affected by AD. This figure will significantly increase to approximately 75.6 million and 135.5 million by 2030 and 2050, respectively. Today, someone develops AD every 67 s in America. According to the center for disease control (CDC), there are now 5.3 million people who have AD in the United States (US), and by 2050, the total number of patients is expected to grow to 13.2 million [1,12,13]. In addition, the World Health Organization (WHO) estimates that 5% of men worldwide and 6% of women over 60 years of age are largely characterized by Alzheimer’s dementia [3,10,14,15]. 

The key pathological features of AD include beta-amyloid (Aβ) plaques, hyperphosphorylated tau proteins, known as neurofibrillary tangles, and neuronal loss, followed by cerebrovascular amyloidosis, inflammation, and other major synaptic changes in the brain [5,6]. AD is a genetically heterogeneous disorder with an early-onset familial form (FAD), which is autosomal dominant and a late-onset sporadic form (SAD) [16]. Mutation in the Aβ precursor protein (APP) and presenilin (PSEN1 and PSEN2) genes are responsible for FAD, while SAD has a complex etiology including genetic, viral, environmental, and metabolic factors, among others. In this regard, apolipoprotein E is a polymorphic protein of three isoforms (APOE2, APOE3, and APOE4) in which APOE4 is the most solid genetic risk factor for SAD [4,9,16,17,18]. Globally, AD has become more and more prevalent among aging populations. In this respect, identification of treatment options will need a better understanding of the pathological mechanisms involved and different ideas for drug design and assessment [19]. Despite the enormous progress made in AD research over the last few decades, the actual cause and pathogenesis of AD are not fully understood, so that there are only a few effective drugs available that can slow the progress rate of the disease [5,12]. Currently, available FDA-approved drugs for the treatment of AD are acetylcholinesterase inhibitors (AChEI) such as rivastigmine, donepezil, and galantamine, and N-methyl-D-aspartate (NMDA) antagonists including memantine. Moreover, just fewer than 20% of patients respond moderately to these drugs with an average betterment in 6 to 12 months. However, approved drugs cannot treat AD without high side effects. There is, therefore, a strong need to identify and evaluate more appropriate medications with fewer side effects. Within this context, numerous herbal molecules have proven effective to prevent and manage various neurodegenerative disorders [12]. Withaferin A (WA), a steroidal glycowithanolide lactone obtained from the Ayurvedic medicinal plant *Withania somnifera* has the potential to be a therapeutic agent for AD. WA is a major biologically active withanolides with a wide range of activities, such as anti-inflammatory, antitumor, anti-angiogenic, and antioxidant activities, and it also inhibits mitochondrial apoptosis [20,21]. According to published research, WA seems to have inhibitory effects against the development of a pathological marker associated with AD. In addition, the underlying protective effect of WA has been studied in a variety of cellular and animal models of AD [22,23,24,25,26,27]. Results indicated that the neuroprotective effects of WA in AD are mediated by its ability to reduce beta-amyloid plaque aggregation, tau protein accumulation, inhibition of acetylcholinesterase (AChE) and butyrylcholinesterase (BuChE) activities, regulation of heat shock proteins, and inhibition of oxidative and inflammatory components. Because there is now no pharmacological therapy that can be efficiently employed for treating AD, the leading hypothesis might be a more recent viewpoint on medication therapy for the disease in the future. Based on the preceding discussion, the present review focuses on the antioxidant, immunomodulatory, anticholinesterase, and anti-inflammatory properties of WA and its derivatives as potential medications for the treatment and prevention of AD [28,29,30].

## 2. Biosynthesis of Withaferin A (WA)

WA is a steroidal lactone belonging to the withanolide group, which was first isolated from the familiar medicinal plant *W. somnifera* in 1965 by Lavie. It is formed from naturally occurring withanolides, which are C-28 steroids with an ergostane-based skeleton functionalized o at C-26 and C-22 to form a δ lactone. Furthermore, the ketone and hydroxyl groups functionally oxidize the C-1 group to form a 1-one [31,32]. WA was also biosynthetically isolated from *Acnistus breviflorus* [33]. Chemoinformatic differentiation sterols, triterpenes, and withanolides indicated that withanolides may have originated from 24-methylene lophenol, lanosterol, campesterol, 24-methylene cholesterol metabolite analogs, or from the triterpene pathway’s upstream intermediates [34]. Although conversion of the 24-methylene cholesterol into withanolides involves a complicated metabolic pathway, which includes glycosylation, hydroxylation, chain elongation, desaturation, cyclization, and epoxidation, that is not fully understood, several researchers have specified the biosynthesis of the responsible gene. However, the triterpenoid pathway occurs by the isoprenogenesis process, which is a biogenetical metabolic course for the determination of isoprene units such as dimethylallyl pyrophosphate and isopentenyl diphosphate [35,36,37,38]. 

Similarly, isoprenogenesis arises in two separate ways in plants. The first is the classical mevalonate (MVA) pathway, which occurs in the cytosol, and the other is the non-mevalonate pathway, also known as the deoxyxylulose pathway (DOXP) or the newly discovered methyl erythreitol pathway (MEP) in plastids [39,40]. In the MVA route, endophyte inoculation up-regulates the expression of HMGR with the concerned isopentenyl pyrophosphate (IPP), which is the predominant pathway [41]. In this case, the enzymes responsible for gene encoding in the transcription process have no effect on 1-deoxy-d-xylulose-5-phosphate reductase (DXR) and 1-deoxy-d-xylulose-5-phosphate synthase (DXS) and may be the primary reason for the maximum productivity of withanolide components [41]. Biosynthetically, isopentenyl diphosphate (IPP) is a five-carbon precursor, a common compound in both MVA and MEP processes [42]. In the MVA pathway, cholesterol is the pertinent starting point for the biosynthesis of withanolides. Acetyl co-enzyme A obtained from activation of acetate is the first step of biotransformation of cholesterol. Two units of acetyl-CoA then convert to mevalonate through metabolization, and by losing one carbon atom, are metamorphosed into isopentenyl pyrophosphate (IPP). It is worth mentioning that the S-form of mevalonate is inert metabolically, while the R-form can produce terpenes in the living system [42].

In contrast, pyruvate integrates with glyceraldehyde-3-phosphate in the MEP pathway, and the presence of DXS enzyme produces 1-deoxy-d-xylulose-5-phosphate (DOXP). Furthermore, DOXP is converted into 2-methylerythritol-4-phosphate (MEP) in the presence of DXR catalyst and finally turns into IPP [42,43,44]. Head-to-tail condensation of IPP with 3,3-dimethylallyl pyrophosphate (DMAPP) produces geranyl pyrophosphate (GPP). Further, farnesyl pyrophosphate (FPP) is obtained by the condensation of trans-geranyl pyrophosphate with another IPP molecule. Similarly, in a head-to-head manner, condensation of two molecules of farnesyl pyrophosphate catalyzed by the enzyme squalene synthase in the presence of NADPH yields squalene [45,46]. In turn, squalene 2,3-epoxide is formed by atmospheric oxygen through an oxidation process that is catalyzed by NADPH-linked oxide. Then, ring closure leads to the formation of lanosterol, which can be converted into a few skeletons of obtusifoliol to steroidal triterpenoid (Delta 8, 14 sterol). 

**Figure 1:**
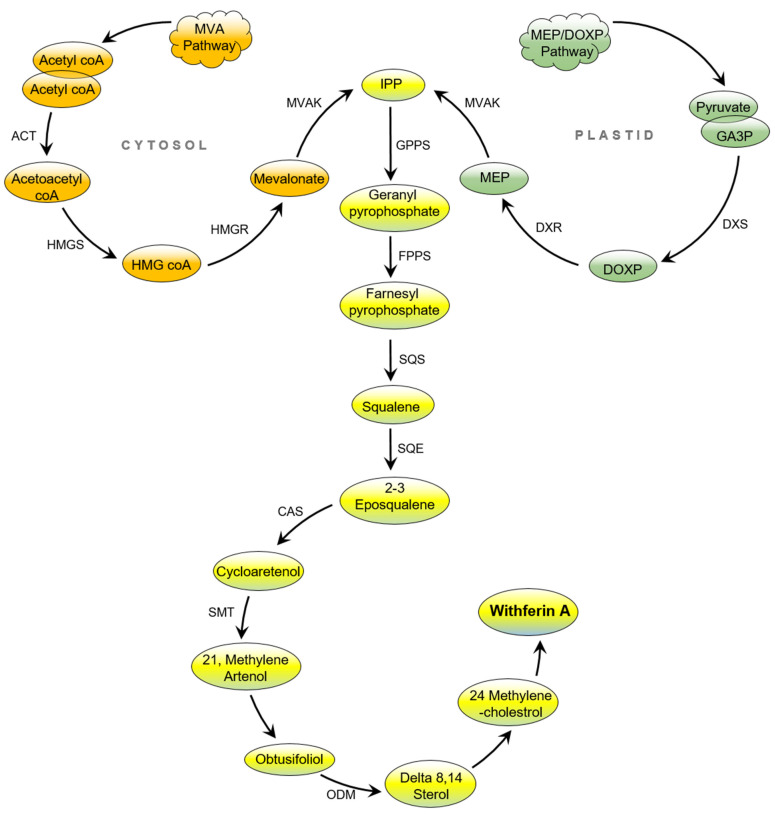
Biosynthesis of WA. Abbreviations: ACT: acetyltransferase; HMGS: hydroxymethyl glutaryl CoA synthase; HMG-CoA: 3-hydroxy-3-methylglutaryl-co enzyme; HMGR: 3-hydroxy-3-methylglutaryl-coenzyme A reductase; MVAK: mevalonate kinase; IPP: 3-isopentenyl pyrophosphate; GPPS: geranyl pyrophosphate synthase; FPPS: farnesyl diphosphate synthase; SQS: squalene synthase; SQE: squalene epoxidase; CAS: cycloartenol synthase; SMT: sterol methyl transferase; ODM: obtusifoliol-14-demethylase; DOXP: deoxy xylulose pathway; MEP: methyl erythreitol pathway; DXS: 1-deoxy-d-xylulose-5-phosphate synthase; DXR: 1-deoxy-d-xylulose-5-phosphate reductase; WA: withaferin A.

Furthermore, the 24-methylenecholesterol biotransformation from lanosterol is not yet fully understood. However, 24-methylenecholestrol may be a biosynthetic precursor to steroidal lactones. In this regard, it has been suggested that WA forms from δ-lactonization between C22 and C26 of 24-methylenecholestrol and the hydroxylation of C22. It has also been proposed that WA is the α,β-unsaturated ketone in ring A of common withanolides and may be produced through the sequence 20–23 [45]. Three sites are prone to Michael addition alkylation reactions and nucleophilic attacks, namely the unsaturated A-ring at C3, the epoxide structure at C5, and the E-ring at the C24 position [47]. Depicted in Figure 1 is the biosynthesis of WA.

## 3. Pathogenesis of AD

AD was first mentioned by Alois Alzheimer at a conference in Germany in 1906 as a “distinctive extreme cerebral cortex disease phase.” Recently, AD is believed to be a persistent or progressive condition, marked by diminished cognitive performance beyond what may be considered the result of normal aging, influencing memory, orientation, thinking, awareness, listening, judgment, and vocabulary [48]. Over a hundred years have passed since the very first pathophysiological dimensions of AD were mentioned. Over the last decades, developments in molecular biology have been critical to the interpretation of the molecular pathways behind AD. Therefore, it is widely understood that the molecular pathogenesis of AD is complex and includes a variety of theories or explanations where several different variables interrelate [49,50]. Nevertheless, neither of these postulates alone is capable of clarifying the whole pathology component, and further research is required [2].

Areas including the original factors of the disorder, the abnormal development of Aβ and the pathways through which it disrupts nicotinic acetylcholine receptors and neurons, and the association between cholinergic disorders and AD cognitive impairment are still not well known [48]. In this context, new findings that lead to the explanation and interaction of AD pathogenic mechanisms are important because they may allow the development of new treatment methods of the disease where current drug therapy cannot inhibit its incidence and growth [2]. Neurobiological pathways underpinning AD have been a key factor in pathological interpretation [51]; specifically, the most popular found alteration can be clarified by the cholinergic hypothesis, the amyloid peptides hypothesis, the tau protein hypothesis, the metal imbalance hypothesis, and the presence of oxidative stress (OS) [2,52].

### 3.1. The Amyloid Hypothesis

John Hardy and David Allsop introduced the amyloid hypothesis in 1991 [4,53]. In this respect, the amyloid β-peptide, which is prevalent in the brain plaques that define Alzheimer’s disease (AD), was decoded from the meningeal blood vessels of AD patients and those with Down syndrome about 20 years ago. The same peptide was shown to be the main component of senile plaques in the brain tissue of an AD patient. These results marked the beginning of the current phase of research into this prevalent, and possibly lethal, neurodegenerative disease [4,53,54]. Amyloid β peptide is a transmembrane protein generated via the amyloidogenic pathway by hydrolysis of the Aβ precursor protein (APP). According to published research, APP generates C-terminal fragments via three routes when hydrolyzed by α-, β-, γ-, and η-secretases [55]. Under normal conditions, the first non-amyloidogenic pathological route results in products that have neurotrophic and neuroprotective properties for nerve cells, such as the soluble ectodomain of APP-α (sAPPα), the C-terminal fragment (CTF)-α, and other smaller fragments, via the participation of α- and γ-secretases. On the other hand, the amyloidogenic pathological pathway is the second route where cleavage of APP by β-secretase results in the formation of CTF-β, whereas cleavage by γ-secretase leads to various lengths of Aβ peptides including Aβ42 [56,57]. In addition, η-secretase regulates the third pathway, which is the alternative processing route under physiological conditions. In this hypothesis, the situation is analyzed as a sequence of anomalies in the development and secretion of the amyloid precursor protein (APP), where the difference between the development and clearance of amyloid β is the starting event and the most important factor responsible for the additional irregularities reported in AD [58]. In this regard, amyloid β is a highly susceptible peptide to proteolytic degradation [59]. Aβ contains 37–43 amino acids, the most common of which are isoforms 1–40 and 1–42 [60,61]. The amyloid peptide isoform 1–42 is the most hydrophobic and has the highest toxicity [1,62]. Due to its physical characteristics, the β-plated sheet is often configured, showing a strong tendency to accumulate and form the amyloid plaque nucleus [58]. It is, therefore, the vital ingredient of neuritic amyloid plaques [2].

#### 3.1.1. Nonamyloidogenic Pathway 

APP is a glycoprotein that is produced in the brain and central nervous system as an integral membrane glycoprotein [63]. APP consists of 770 amino acids, where Aβ includes 28 residues just outside the membrane in addition to 14 residues from the APP transmembrane domain [64]. It can be processed sequentially by two proteolytic pathways, namely the non-amyloidogenic and the amyloidogenic route [63]. The non-amyloidogenic route leads α secretase-mediated cleavage of full-length APP. The cleavage removes the sAPP ectodomain from the cell membrane and leaves an 83-amino-acid-long C-terminal APP fragment (α-CTF) inside the plasma membrane. The α-CTF will then be cleaved further by γ-secretase, releasing a tiny p3 fragment into the extracellular area while the rest of APP intracellular domain remains in the cytoplasm [65]. The major α-secretases are ADAM10 and ADAM17, which are the metalloproteases in neurons. A small p3 hydrophobic fragment is generated by α-secretase, which plays a role in normal synaptic signaling and is further sliced at residue 711 by γ-secretase; however, its exact functions still need to be clarified. Endocytosis of cell-surface APP leads to endosome growth of Aβ that contributes to extracellular discharge and accumulation of Aβ [66].

#### 3.1.2. Amyloidogenic Pathway

The amyloidogenic process involves the proteolytic cleavage of APP by β-secretase (BACE1) and the γ-secretase complex in a sequential manner [63,67]. In this regard, BACE1 is a membrane-spanning aspartyl protease [63], whereas γ-secretase is an intramembrane aspartyl protease composed of four proteins: PEN2, nicastrin, presenilin, and anterior pharynx defective 1 (APH-1) complexed together [68,69]. γ-Secretase converts the complex into insoluble and neurotoxic Aβ fragments [70]. The first and rate-limiting step in this process is the β-secretase cleavage [66,71]. β-Cleavage releases the sAPP ectodomain, and a 99 amino acid APP carboxy-terminal fragment (C99) can be cleaved further at numerous sites by γ-secretase. APP cleavage by γ-secretase can result in amyloid peptides with varying chain lengths, such as Aβ37, 38, 39, 40, 42, and 43 [72]. These peptides form Aβ oligomers, whereas aggregated plaques are produced by further polymerization [63]. In this respect, Aβ42 and Aβ40 are the two primary Aβ species found in the brain. Although soluble, Aβ40 is substantially more prevalent than soluble Aβ42; Aβ42 has a greater tendency for aggregation because of the hydrophobicity within its two terminal residues. In addition, Aβ42 is the key element of amyloid plaques, which has been found to be neurotoxic [66,73,74]. As a result, Aβ42 is assumed to be a critical participant in plaque development and AD pathogenesis [75]. Furthermore, it has been demonstrated that the levels of Aβ38, Aβ42, and the Aβ42/Aβ38 ratio in cerebral spinal fluid may be utilized to differentiate AD from other dementias [3,76]. On the other hand, fibrillar Aβ has been demonstrated to enhance cytokine and nitric oxide generation in CD14, TLR2, and TLR4-dependent microglia [77,78]. Stewart et al. discovered yet another signaling mechanism via which Aβ may cause inflammation [79]. These authors discovered that Aβ causes inflammatory signaling by forming heterodimers of Toll-like receptors 4 and 6. Interaction of Aβ with the scavenger receptor CD36 regulates the assembly of this heterodimer. In this context, participation of CD14, CD36, and Toll-like receptors in Aβ-induced microglia activation clearly demonstrates that innate immunity is associated with AD pathogenesis [80,81].

### 3.2. The Tau (τ) Hypothesis

The amyloid cascade concept has been the prevailing hypothesis in AD research, although it does not fully explain the etiopathogenesis of the disease. According to this hypothesis, the τ protein appears as a secondary pathogenic event that leads to neurodegeneration [2,82]. Tau is a microtubule-associated protein that is generated via the MAPT gene’s alternative splicing [83]. It is mostly present in neuronal axons of the brain where it interacts with microtubules (MTs) [84]. Tau was first separated from plaques in the brains of AD patients by Claude Wischik in 1988 and demonstrated for the first time that it may be the cause of dementia [58,85]. It is a highly soluble protein related to microtubules, and its role is to stabilize microtubules [86]. These microtubules provide support for axonal transport, structural improvement, and neuronal development. In normal circumstances, Tau has a few phosphorylation sites and adversely regulates the attachment of τ to microtubules. However, tau phosphorylation can become saturated under pathological conditions. Development of tau pathology is a complex multifactorial process, and dysfunctions arise in the phosphorylation mechanism of the τ protein in AD, resulting in the enzyme’s hyperphosphorylation. In the brains of AD patients, hyperphosphorylated tau induces configuration alterations and tubulin polymerization capacity reduction [87], culminating in microtubule dysfunction [88]. Increased cytosolic tau levels cause tau–tau interactions and polymerization, resulting in insoluble PHFs and straight filaments (SFs), which form intraneuronal fibrillary deposits called neurofibrillary tangles (NFTs) [87]. In this respect, AD is often distinguished by the inclusion of NFTs and hyperphosphorylation of the microtubule-associated τ proteins that form these tangles [89]. NFTs are fragments of combined, as well as helical, protein filaments in neuron cytoplasm cells [63]. When the τ protein interacts with kinases produced as a result of the increasing amount of Aβ in the system, it is hyperphosphorylated [86]. Due to the separation of the subunits of the tubules, the tubule becomes unstable, breaks apart, and then turns into large parts of the τ filaments, which are further consolidated into NFTs [63]. Such NFTs are fibrillary, smooth, and highly insoluble patches in neuronal cytoplasms and systems, due to abnormal neuron connectivity, signal processing, and, finally, neuron apoptosis [58,90]. Research findings indicated that cleavage and phosphorylation of τ are regulated by soluble Aβ for the generation of NFT [66,71]. In summary, the pathophysiology of pathogenic tau is still clearly unknown due to its intricacy and needs further study [91]. 

### 3.3. Oxidative Stress Hypothesis

Both normal and pathological processes in the human body produce reactive oxygen and nitrogen species (ROS and RNS). The brain is more vulnerable to oxidative stress (OS), as it requires 20% more oxygen than other mitochondrial respiratory tissues, so that the intake of oxygen in the brain is high [92]. It has long been understood that the association between neuronal apoptosis and OS is one of the common features of neurodegenerative diseases [93]. ROS interact chemically with biological molecules including lipids and proteins, nucleic acids, and cell organelles [50,94]. Moreover, the known pathology of senile plaques and neurofibrillary tangles in the presence of substantial OS is typical of the AD brain [95]. Accumulation of damaging free radicals and alteration of the action of antioxidant enzymes such as superoxide catalase and dismutase are also found in AD. In AD pathogenesis, OS is an essential factor, but the causes of abnormal free radicals production and the mechanisms by which redox equilibrium is changed are not clearly understood [67,96]. Published research showed that abnormal aggregation of amyloid β can assist the generation of ROS via a process involving activation of NMDA receptors, and that OS can increase the development and aggregation of amyloid β along with the promotion of τ phosphorylation and polymerization, producing a vicious circle that facilitates the development and progression of AD [97]. 

### 3.4. The Cholinergic Hypothesis

The cholinergic hypothesis was the first molecular approach to the pathophysiology of AD. This hypothesis states that AD is a primary degenerative process capable of selectively damaging groups of cholinergic neurons in the amygdala, hippocampus, nucleus basalis, frontal cortex, and medial septum regions, which play important roles in attention, awareness, learning, memory, and other mnemonic processes [59]. Furthermore, ROS may be responsible for the formation of Aβ plaques, resulting in a decrease in the production of the cholinergic neurotransmitter acetylcholine (Ach) in the cortical and basal ganglia of the brain. This might alter synaptic communication, which may lead to inflammatory processes, memory loss, and cell death [23]. ACh is immediately hydrolyzed after discharge at the synapse by AChE to prevent the negative consequences of excessive cholinergic stimulation. On the other hand, BuChE is a related enzyme with the same functionality. ACh does not hydrolyze as fast when cholinesterase is blocked, and ACh levels increase [2,98]. As a result, AchE and BuChE inhibitors are potential targets for AD. 

Interestingly, apolipoproteins are major players in regulating Aβ pathology. The ApoE gene encodes three alleles, namely ApoE2, ApoE3, and ApoE4It, where ApoE4 is the major genetic risk factor for sporadic AD and is associated with cognitive deficits [99]. The effect of ApoE on AD was studied in the context of the “cholinergic hypothesis” in 1976, by recognizing that cholinergic neurons are not the crucial target of AD. In specific areas of the brain, cholinergic receptor binding decreases with minor to severe AD, which is associated with neuropsychiatric symptoms [59]. In vivo binding of the cholinergic receptor may reveal links to other key changes in the brain connected with aging and AD and may provide a potential target for molecular treatment. In this case, a significant cholinergic neuron loss in the forebrain nucleus leads to a decrease in the acetylcholine-mediated neurotransmission. In addition, drugs that contribute to the regulation of acetylcholine transmitter levels, such as donepezil and cholinesterase inhibitors (ChEIs) have laid the foundation for symptomatic AD therapy for more than 20 years [10].

### 3.5. Inflammatory Hypothesis 

Advances in the field of AD research seem to indicate that the pathogenesis of the illness does not exist only in the neuronal compartment but has a significant interplay with immune systems in the brain [100]. Neuroinflammation in AD is not a passive system triggered with the emergence of senile plaques and neurofibrillary tangles, but rather is due as much to pathogenesis as plaques and tangles themselves [101]. Numerous findings indicating genes encoding immune receptors, such as TREM22 and CD33, linked to AD, highlight the importance of neuroinflammation [102,103]. A critical step in AD is believed to be the ability of microglia to attach to soluble Aβ oligomers and fibrils through cell-surface receptors such as α6β1 integrin, CD36, CD14, SCARA1, CD47, and Toll-like receptors, namely TLR2, TLR4, TLR6, and TLR9 [79,104,105]. Activation of microglia leads to the production of proinflammatory cytokines and chemokines, which cause binding of Aβ with CD36, TLR4, and TLR6 [79,106,107]. In this respect, genetic deletion of these three genes (CD36, TLR4, and TLR6) limits Aβ-induced cytokine production in vitro and blocks the aggregation of amyloid proteins and the activation of inflammasomes [107,108,109]. Following the binding of Aβ to this receptor complex leads to intracellular signaling results in the signal transduction of the nuclear factor-κB (NF-κB) from the cytoplasm to the nucleus [110]. In addition, this leads to the initiation of protein kinase A, response element-binding protein [111,112], which results in the transcription of numerous pro-inflammatory cytokines, cyclooxygenase-2 (COX-2), and NO-synthase [111,113]. Recently, Aβ has been reported to induce NLRP3 inflammasome, high-molecular-weight protein complexes that are involved in several inflammatory pathophysiologies in microglial cells in vitro and in vivo, thus identifying a novel mechanism that could contribute to the development of AD [111,112]. As a result, stimulation of NLRP3 inflammasome by Aβ in microglia is essential for IL-1β maturation and subsequent inflammatory events [114]. Increasing our understanding of the ways that these immune processes affect AD might lead to future therapeutic or preventative approaches.

## 4. Epidemiology and Clinical Epidemiology

AD is a slowly progressive neurodegenerative disease. In the USA, there were approximately 4.5 million persons with AD in 2000. However, as the age of the population in the next 50 years increases, the number of persons who will have AD will increase by threefold in the next 50 years, and there will be about 13.2 AD cases in 2050 [115]. In this respect, age and gender are the potential risk factor for AD. Statistics showed that AD cases were 66/100,000 per year at age 60–69, 409/100,000 per year at age 70–79, and 1479/100,000 per year age 80 and older in 1960–1975. In 2014, WHO reported that 4000 Alzheimer’s death annually occurred in Iran [115]. The epidemiological study of AD with other risk factors of AD was conducted in five cities of Khuzestan Province in 2016–2018. Results indicated that smoking has a significant role in AD, where 26.9% of Alzheimer’s patients had a history of smoking, 34.8% a history of diabetes, and 26.3% had Alzheimer’s family history [116]. Additionally, research findings showed that women have a higher risk of AD than men. Statistics revealed that AD has a prevalence of approximately 1 percent among those 65 to 69 years of age, and increases 40 to 50 percent among persons 95 years of age and over [117]. Finally, family history is one of the significant risk factors for AD. In this regard, when family history is compared with age in AD patients, about 1% developed AD by age; in contrast, 20% developed their AD from their family [118].

### Clinical Epidemiology

In the last 30 years, the criteria for AD have been changed in several circumstances; however, neuropathologic diagnosis has been considered as the gold standard. In this respect, the National Institute on Aging (NIA)-sponsored Alzheimer disease centers (ADCs) collected the accuracy rate of AD from 2005 to 2010. Data revealed that the clinical diagnosis of AD with other dementia varied depending on several criteria, which were conducted by epidemiologic studies, clinical trials, and governmental healthcare analyses [118]. Clinical studies on 1075 Yoruba population members of Ibadan in Iran demonstrated that with increasing levels of cholesterol, APOE-ε4 increased the risk of AD [119]. In another study, the amplitude of AD was investigated depending on age. Results revealed that 10.3% of those over 65 years of age had AD. Additionally, results showed that 3% of those aged 65–74, 18.7% of those aged 75–84, and 47.2% of those over 85 years of age had AD [116]. 

## 5. Therapeutic Implications of WA in AD

WA is a secondary metabolite that is used to treat AD. An in vitro study demonstrated that *W. somnifera* root contains WA, which has a significant effect on H_2_O_2_ and Aβ amyloid-induced neurotoxicity [120]. In an AD model, cognitive defects induced by ibotenic acid that was significantly reversed by WA isolated from Ashwagandha root [121]. Oral administration of WA to mice caused a boost of AChE activity with improving muscarinic receptor binding abilities in the cortical area but did not affect the GABA, benzodiazepine, NMDA, or AMPA receptor in the globus pallidus and the lateral septum area of the brain. In contrast, WA reversed cholinergic marker reduction and enhanced binding affinity with the muscarinic receptor, suggesting that WA could be a potential compound for AD therapy [122]. On the other hand, the pro-inflammatory mediator iNOS (a) moderated Aβ nitration that ameliorated peptides inclination to aggregate nitration of Aβ, (b) enhanced the peptide’s propensity to formed seeding core and aggregated Aβ plaques, (c) inhibited iNOS expression and NF-κB signaling pathway, and (d) diminished aggregation of Aβ plaques in the cerebral area and flourished cognitive performance [123,124].

Similarly, WA inhibited NF-κB and iNOS markers in astrocytes [125]. Furthermore, Hsp90 accelerates aberrant accumulation and aggregation of τ protein [126] and induces Aβ toxicity in AD [126]. In addition, WA inhibited Hsp90 [127] and induced Hsp 27 and Hsp70 expressions, and reduced τ aggregation in a mouse model of tauopathies [128,129]. Cholinesterase enzyme develops AD, which was inhibited by WA obtained from the water extract of Ashwagandha [130]. It inhibited nuclear factor NF-κB activation, which is responsible for the expression of oxidation and inflammation gene, thus prevented the accumulation of Aβ. WA also increased the neuro-protective protein heme oxygenase-1, which is beneficial to AD prevention [131,132]. WA additionally enhances memory [133], prevents Aβ production, reconstructs synapses, and regenerates axons [134]. AD is often related to the hyperactivity of microglial cells. In this respect, WA modulated their action to prevent brain tissue damage [135]. In this respect, an in vitro study demonstrated that WA inhibits Aβ production in microglial and SH-SY5Y cells overexpressing amyloid precursor protein (SH-APP cells) [135] and suppresses NF-κB mediated neuro-inflammation [136]. Multidomain protein LRRK2 remains often mutated, which elevates kinase expression to enhance AD, regulated by WA [137,138]. Berghe et al. (2012) demonstrated that the TAR DNA-binding protein-43, a pathological hallmark of AD, is alleviated by WA and boosted autophagic marker LC3BII level in the brain, while it suppressed NF-κB-dependent neuro-inflammation in frontotemporal lobar degeneration (FTLD) in a mice model [27].

### 5.1. Role of WA and Its Derivatives

WA is a C-28-steroidal lactone withanolide that is found in *W. somnifera* [139,140]. WA and its derivatives, at the molecular level, extracted from *W. somnifera* root, exhibit beneficial effects in AD by blocking Aβ production, inhibiting NF-κB activation, preserving synaptic function, decreasing apoptotic cell death, reversing the reduction in cholinergic markers, and improving antioxidant effects via the migration of Nrf2 to the nucleus. Similarly, Nrf2 improves the activation of antioxidant enzymes. It is recommended that the Nrf2 translocation to the nucleus is triggered by WA, where the transcription factor increases the expression of neuroprotective heme oxygenase-1 proteins [141]. The benefits of *W. somnifera* root constituents, especially WA in neurodegenerative diseases, could be attributed to their anti-inflammation, neuritis-promoting, anti-apoptotic, antioxidant, and anxiolytic activities, and also to their capability to restore energy levels, mitochondrial dysfunction, and increase antioxidant defenses like reduction of glutathione [141,142]. The possible underlying mechanism of the neuroprotective activities of *W. somnifera* could be controlled through several pathways. In this respect, studies have indicated the neuroprotective effect of WA [143,144], withanolide A [110], and other derivatives along with the synergistic action of multiple compounds that contribute towards the beneficial effect in the pathogenesis of neurodegenerative diseases [21].

#### 5.1.1. WA and Its Derivatives Aβ Plaque Formation Inhibitors

Published research indicated that Aβ deposition in the brain is one of the main factors in neurodegeneration [145]. As a result, amyloid β is one of the therapeutic targets for the development of possible AD treatments [146]. By limiting the production of this peptide, compounds that may aid in their accumulation and aggregation are sought for both the prevention and advancement of AD. However, research findings showed that these strategies have failed to provide therapeutic benefits in large clinical trials for those with mild to severe AD. As a result, molecular species called Aβ oligomers are currently the primary focus, and numerous monoclonal antibodies have provided promising outcomes when directed against these species [147]. Because Aβ oligomers are in equilibrium with both monomeric and aggregated species, earlier medications that effectively eliminated monomeric Aβ or Aβ plaques should have shown therapeutic advantages as well.

Tiwari and coworkers conducted the first study, which showed that WA could protect the brain from the toxic effects of amyloid-beta plaques. These researchers demonstrated that amyloid-beta severely affects neuronal functions and structures, and WA decreased the secretion of amyloid β and induced neurotoxicity in SH-SY5Y cells (SH-APP) in which the amyloid precursor protein (APP)-plasmid was transfected. When SH-APP cells were exposed to neurotoxic HIV-1 Tat and cocaine, the level of Aβ40 rose. Both neurotoxic protein HIV-1 Tat-induced and cocaine-induced Aβ aggregates in SH-APP were significantly reduced by employing WA with a concentration of 2 μM. Additionally, WA diminishes the amount of beading on the cell membrane and the occurrence of cytoplasmic vacuoles, two indices of neurotoxicity. However, further research is required to prove WA effectiveness in vivo [24]. A follow-up investigation examined the role of WA on Aβ formation by using congo red (CR) staining to highlight the cells. Results showed that WA treated SH-APP cell cultures display less staining with the toxic Aβ peptide than dimethyl sulfoxide (DMSO)-treated SH-APP cell cultures. DMSO-treated SH-APP cells expressed larger quantities of Aβ, which is stained red after being CR tagged. As SH-APP cells treated with WA had lower amounts of Aβ, there was less staining in these WA-treated cells [148].

Published work demonstrated that derivatives such as withanolide A increases α-secretase expression and decrease β-secretase expression in cultured normal rat cortical neurons. When APP is treated with ADAM10, production of Aβ is reduced due to alternative production of soluble and non-toxic APPα. As a result, withanolide A enhanced production of soluble APPα in cultured neurons. It was also shown that withanolide A enhances the expression of a significant proteolytic insulin-degrading enzyme (IDE), which is associated with Aβ degradation [4,146,149,150]. These findings indicate that withanolide A may decrease Aβ by enhancing the production of soluble APPα and Aβ. In light of these reports, withanolide A is a significant candidate for a multifunctional Alzheimer’s disease drug [4,146,149,150]. Furthermore, neuronal cell death caused by amyloid plaques was blocked by withanamide. Molecular modeling studies have shown that withanamide A and C attach uniquely to the active beta-amyloid pattern (Aβ 25–35) and block fibril formation [7,151].

#### 5.1.2. WA Acts as Antioxidant in AD

AD etiology often starts with the accumulation of β-amyloid fibrils, accompanied by tau pathology and then neuronal cell death. In this respect, the free radical-triggered harm of the brain tissues is also one of the vital factors considered in this disease. Furthermore, increased levels of Al, Fe, Hg, and Ca decreased polyunsaturated fatty acids, increased oxidation of protein and DNA and lipid peroxidation, and the presence of oxidation products such as carbonyls, malondialdehyde, heme oxygenase-1, and peroxynitrite, which are initiators of free radicals, could trigger AD [146].

In addition, WA was evaluated for antioxidant activity using major free-radical scavenging enzymes such as catalase (CAT), superoxide dismutase (SOD), glutathione peroxidase (GPX), and NADPH dehydrogenase levels in the frontal cortex and striatum of the rat brain [28]. It exhibits anti-oxidative activity by increasing the levels of various antioxidant enzymes, which reduce the free radicals or stress formed in the affected cells and provide a healthy mitochondrial function. This function is demonstrated in several recent studies by simple testing of treated and untreated mouse models and human cell lines. Treated cells with WA have significantly reduced cellular oxidative damage [152,153]. Accordingly, these findings indicate that *W. somnifera* extracts, especially WA, reduce oxidative stress and mitochondrial dysfunction, and their beneficial role appears to be primarily based on their antioxidant properties [6]. Withanamides are a subset of these components that have been proven to scavenge free radicals produced at the time of the initiation and progression of AD [7,151].

#### 5.1.3. WA Inhibits AChE and BuChE Activities

The first unsuccessful research that investigated cholinesterase inhibition laid the groundwork for other treatments that were developed based on cholinesterase inhibition [154]. The current treatment of AD comprises drugs such as AChE inhibitors and N-methyl-D-aspartate receptor antagonists [155]. It is important to note that AChE inhibitors only temporarily relieve some of the disease’s cognitive symptoms and do not stop the patient’s cognitive loss. According to a literature assessment, the efficacy of conventional medications is statistically substantial, but clinical relevance is restricted to disease slowing rather than reversal [156]. In addition, adverse effects such as disorientation, falls, dizziness, and fatigue may occur with these medications and should be used only as recommended [156]. The limited clinical effectiveness of existing AChE inhibitors and N-methyl-D-aspartate (NMDA) receptor antagonists has sparked a search for therapeutic approaches that target the causes of neurodegeneration that are closer to the source.

Within this context, WA exhibited beneficial properties for the prevention of AD. WA has an important role in AD by reversing the reduction in cholinergic markers such as choline acetyltransferase (ChAT) and acetylcholine [28,150,151]. In an in vitro assay, the AChE and BuChE enzyme activity was blocked by WA [21]. Based on sub-acute toxicity findings, *Withania somnifera* extract (containing 4.5% of WA) up to a dose of 2000 mg/kg/day did not cause adverse effects when administered orally [157]. These actions of WA could illustrate the well-known cognition enhancement effects due to preferential activity on cholinergic neurotransmission to the brain areas like the basal and cortical forebrain, which is involved in cognitive function [122,153]. It is, however, unknown how this effect occurs, although it may be related to the way cholinergic neurotransmission is controlled. The neurotransmitter systems in the brain were monitored after the administration of an extract containing WA. Findings demonstrated that the extract increases cholinesterase activity in the globus pallidus and lateral septum parts of the brain and enhances muscarinic M1 receptor binding in cortical regions. Additionally, WA increased the level of ACh, the amount of choline acetyltransferase (ChAT), and other cholinergic indicators such as choline acetyltransferase (ChAT) in rats [158]. The preferred influence on cholinergic neurotransmission in the cortical and basal forebrain, brain regions that are important in cognitive function, may be able to explain the reported cognition-enhancing benefits of WA. Another advantage of WA is that it has neither effect on GABAA, NMDA, or glutamate receptor subtypes, nor does it interact with benzodiazepine receptors [159]. WA might have future value in the treatment of AD, but additional research is needed to confirm this assumption [98,158,159].

On the other hand, the neuroprotective potential of WA (50 mg/kg b.w.) revealed a recurrence of homo vanillic acid (HVA) and dopamine (DA) in striatum and substantia nigra. Reduction of these catecholamines results in motor deficits. The neuroprotective potential of WA is also indicated by the elevated level of DA and HVA in the treatment of AD [21]. Within this context, a powerful neurotoxicant, ibotenic acid, has been used to study the effect of brain lesions. When it is injected into the brains of rats, mice, or monkeys, it results in reflective destruction of the basal cholinergic neurons of the forebrain. Recent studies showed that WA and sitoindosides significantly reverse ibotenic acid-induced cognitive impairment in an AD model [15,17,28,152,160,161].

#### 5.1.4. WA Blocks the Neuroinflammation in AD

**Figure 2:**
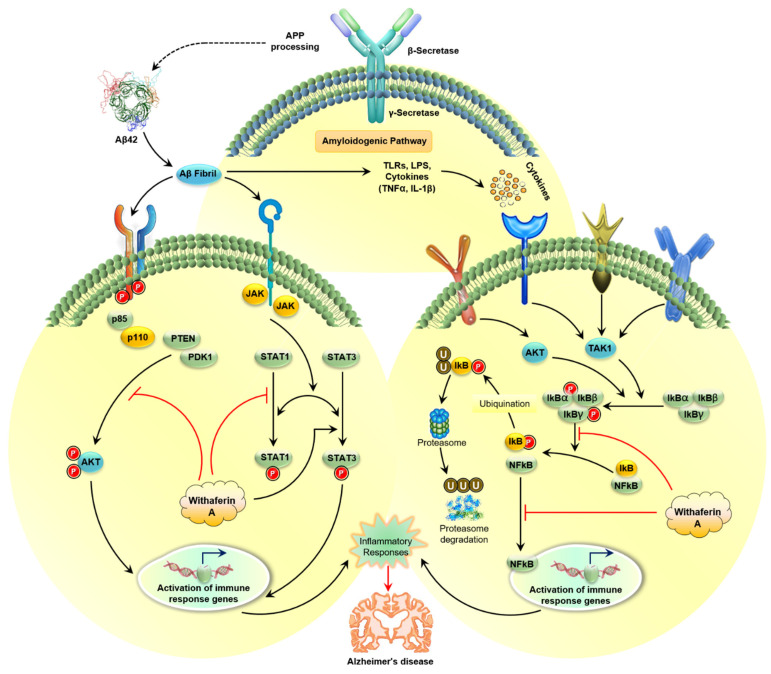
Schematic representation showing the target sites of WA action in amyloidogenic pathway that leads to AD. WA inhibits NF-κB signaling (right side). WA regulates several kinase-signaling pathways such as AKT and JAK/STAT. Abbreviations: APP: amyloid precursor protein; Aβ: β-amyloid; TLR: Toll-like receptor; LPS: lipopolysaccharides; IKK: IκB kinase; TNF-α: tumor necrosis factor-α; IL-1β: interleukin-1β; MAP3K7)/TAK1: mitogen-activated protein kinase 7 (MAP3K7)/(TAK1); NF-κB: nuclear factor kappa B; U: ubiquinone; JAK: Janus kinase; STAT: signal transducers and activators of transcription; PTEN: phosphatase and tensin homolog; PDK1: phosphoinositide-dependent kinase-1; AD: Alzheimer disease; WA: withaferin A.

In AD, microglia activation is achieved through the interaction between Aβ fibrils and Aβ oligomers that initiate inflammatory reactions by promoting NLRP3 inflammasome and nuclear factor NF-κB, which is responsible for the release of pro-inflammatory chemokines and cytokines [114]. Microglia regulate Aβ fibrils by the process of phagocytosis, and then neprilysin and insulin-degrading enzymes destroy these fibrils. In AD, the NF-κB pathway and expression of the NLRP3 block the phagocytosis of Aβ fibrils, which results in increasing the buildup of Aβ fibrils in the brain, creating a self-perpetuating loop and finally leads to neuroinflammation [114]. In addition, WA inhibits the expression of NF-κB, which plays an important part in the cascade of inflammatory cytokines. Additionally, reduced expression of STAT and JAK, and increased expression of IKBKG and IKBKB have been observed (Figure 2) [162]. By targeting critical inflammatory pathways like NF-κB and nuclear factor erythroid 2 related factors 2 signaling, WA defeats inflammatory disease in numerous in vitro and preclinical in vivo models of neurodegenerative disorders like AD and PD, cystic fibrosis, and osteoarthritis (Table 1) [20].

##### WA Inhibits the NF-κB Pathway

In normal and pathophysiological immune responses, the transcription factor NF-κB and its activation signaling pathways play a critical role, making it an interesting therapeutic target. Therefore, WZA has been extensively studied to investigate its effect on the signaling NF-κB activation [163,164,165]. 

WA decreases inflammatory mediators including TNF-α and IL-1 in plaque formation and neurodegeneration [152,161,166,167]. *W. somnifera* is anti-inflammatory due to the presence of WA. Research findings indicated that WA effectively prevents the activation of NF-κB by stopping phosphorylation and deterioration by inhibiting stimulation of IκB kinase, while other *W. somnifera* derived steroidal lactones, like withanolide A, are far less effective [168]. Moreover, WA inhibits NF-κB activation by attacking cysteine 179 located at the IKKβ catalytic site [165]. Furthermore, nitric oxide and COX-2 production are inhibited by WA by inhibiting nitric oxide synthase (iNOS) [152]. WA also inhibits vascular cell adhesion molecule (VCAM)-1 and intracellular adhesion molecule (ICAM)-1, which is induced by TNF-α along with inducible iNOS expression and NO production induced by lipopolysaccharide (LPS) by reducing Akt and NF-κB activation [169]. Moreover, WA inhibits microglial inflammatory response through inhibition of LPS-induced COX-2 mRNA and protein expression and production of prostaglandin E2, one of the most important products of COX-2. As a result, WA controls the microglial inflammatory response by decreasing COX-2 expression and PGE2 construction and blocking LPS-induced STAT1 and STAT3 phosphorylation [170]. There were no significant effects of WA on LPS-induced ERK and Akt phosphorylation, but WA reduced slightly the JNK-pathway and p38 phosphorylation (Figure 2). 

Several molecular docking studies also revealed that WA is capable of interfering with the NF-κB production pathway through different mechanisms. Among these mechanisms is in silico analysis, which has shown a solid probable intermolecular interaction between WA and IKKγ that interrupts the development of the IKK complex and inhibits IκB depletion [171]. Another suggested mechanism of WA-mediated inhibition of NF-κB is by straight interaction of WA with NF-κB itself [172] or its IκB inhibitor [20,172]. Thus, it was concluded that pure WA has important NF-κB inhibitor activity, which makes it a novel anti-inflammatory agent for the treatment of AD [163].

##### WA Affects Inflammasome Activation

**Figure 3:**
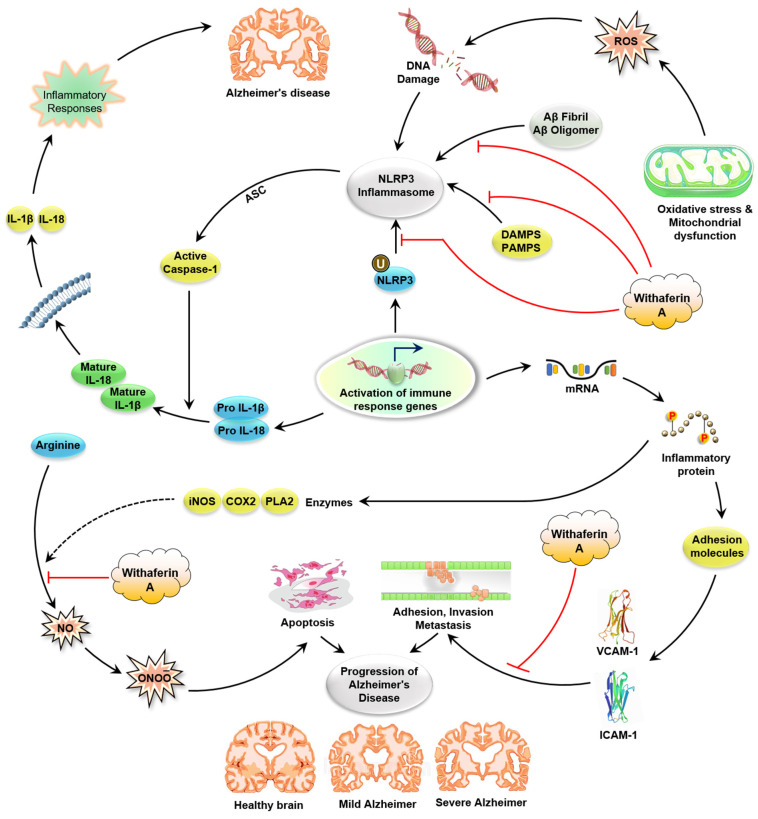
Overview of inflammatory signaling pathways altered by WA through direct molecular targets. Fibrillary Aβ, oxidative stress, DAMP, and PAMP can contribute to the activation of the inflammasome. Aβ fibrils trigger the activation of microglial cells and thus give signal 1 via NF-κB transcription of pro IL-1β and NLRP3. Intracellular aggregation of soluble Aβ and lysosomal rupture by phagocytosis Aβ fibrils may perform another signal, and oxidative stress contributes to the formation of an active NLRP3 inflammasome. Active caspase-1, released from active NLRP3, converts IL-1β pro to active IL-1β, which is released into extracellular space and leads to neuroinflammation and finally AD. WA prevents NLRP3 inflammasome formation and activation by blocking several steps of this pathway. Abbreviations: ROS: reactive oxygen species; NLEP3: NOD-like receptor protein 3; DAMP: damage-associated molecular pattern; PAMP: pathogen-associated molecular pattern; COX-2: cyclooxygenase-2; IκB: inhibitory subunit of NF-κB; IL-18: interleukin-18; VCAM-1: vascular cell adhesion molecule 1; ICAM-1: intercellular adhesion molecule 1; AD: Alzheimer disease; WA: withaferin A.

Inflammasomes, a collection of multiprotein complexes, are gathered inside the cytosol after sensing pattern-associated molecular patterns (PAMPs) or danger-associated molecular patterns (DAMPs) [173]. Once gathered, they function as a scaffold for recruitment of the inactive pro-caspase-1 zymogen that contributes to the oligomerization of the pro-caspase-1 protein and triggers their autoproteolytic cleavage into the bioactive enzyme caspase-1. The corresponding cleavage of pro-IL-18 and pro-IL-1β allows the discharge of such pro-inflammatory cytokines into the extracellular space. In this respect, two main groups of inflammasomes have been recognized based on the presence of their intracellular receptors, AIM2-like receptor inflammasomes (ALR) and Nod-like receptor inflammasomes (NLR) [174]. Numerous studies using inflammatory disease models showed that WA interferes with the development of a crucial subtype of the NLR class inflammasome NLRP3 (Figure 3). Stimulation of the NLRP3 inflammasome is induced by protein aggregations such as β-amyloids, toxins, pathogens, and crystals [175,176,177]. Abnormal activation of NLRP3 has been known to be involved in the initiation and progression of inflammation [178]. Though the precise biological mechanism of WA-mediated blocking of NLRP3 inflammasome development needs further exploration, medicinal use of WA may provide a chance to control cytokine activation in inflammatory diseases [177]. It is essential to know that the impact of WA on other inflammasome subtypes also needs to be examined, since some studies have shown that WA treatment increases the expression and initiation of AIM2 inflammasomes of M2 macrophages, resulting in a pro-inflammatory instead of the anti-inflammatory response in immune cells [20,179].

##### WA Regulates Heat Shock Proteins

WA may control the activity of heat shock proteins (HSPs) that are highly conserved molecular chaperones associated with the transporting, folding, maintenance, and assembly of main regulatory protein kinases [180,181]. In addition, WA controls the target proteins of HSP90 such as AKT and IKKK-complex by attacking and disassociating the CDC37–HSP90 complex, by either preventing the protein cleavage of CDC37 [182] or by straight binding of HSP90 itself. The therapeutic significance of WA in CDC37–HSP90 blockage has already been proven in N9 microglial cells, where one specific target of HSP90 kinase is significantly reduced [182]. On the other hand, LRRK2 is a multi-domain unidentified protein often mutated in patients with sporadic and familial AD. Most mutations in this protein kinase are linked to functional activities such as enhanced protein expression, which is essential for the etiology of inflammatory diseases [20]. Interestingly, a more helpful anti-inflammatory function of WA was proven in TAR–DNA binding protein transgenic mice (TDP43) [183]. Altogether, these data verify that WA has tremendous potential as a natural neurotherapeutic agent for improving cognitive deficits associated with AD. Thus, its application in other neurodegenerative disease models needs to be investigated [21].

**Table 1:** Preclinical trials of WA.

Plant Extract	Method	Subject	WA Mediated Protective Effect	Possible Mechanism	Up/Down Regulation	References
Aqueous methanol extract of *Withania somnifera* roots	In vivo	Mice	Reversed anti-AChE activity	Enhance ACh, choline acetyltransferase; ChAT activity in globus pallidus and lateral septum	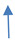	[22]
Aqueous chloroform extract of *Withania somnifera* roots	In vivo	Rat	Anti-cholinergic activity	Reduced cholinergic marker activity	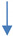	[23]
*Withania somnifera* root extract	In vitro	SH-SY5Y cells (SHAPP)	Anti-amyloidogenic	Aβ40	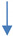	[24]
*Withania somnifera* root extract	In vitro	SHAPP cells and CHME5 microglial cell line	Anti-inflammatory	NF-κB, JUN and STAT gene, IL-1β	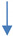	[24,148]
*Withania somnifera* extract	In vitro	SK-N-SH cells	Anti-oxidant and anti-cholinergic	ROS, Ache, Aβ peptide toxicity	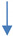	[25]
Aqueous root extract of *Withania somnifera*	In vitro	Rat pheochromocytoma (PC12) cell line	Anti-Alzheimer activity	H_2_O_2_- and Aβ-induced toxicity	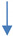	[26]
Plant extract of *withania somnifera*	In vivo	Male Wistar rats	Anti-Alzheimer activity	Reduced acetyl cholinesterase	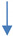	[27]
Plant extract of *withania somnifera*	In vitro	Amyloid-β marker thioflavin-T	Anti-amyloidogenic	Reduced amyloid beta	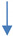	[184]
WA	In vivo	HFD-induced obese mice	Anti-obesity	COX2, NF-κB, TNF-α, inflammation, oxidative stress, and insulin resistance	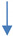	[185]
WA	In vitro, in vivo	Human umbilical vein endothelial cells (HUVECs), mouse	Anti-inflammatory	Inhibit phorbol-12-myristate-3-acetate (PMA), TNF-a, (IL)-1β, PMA-stimulated phosphorylation of p38, extracellular regulated kinases (ERK)-1/2, and c-Jun N-terminal kinase (JNK)	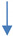	[186]
WA	In vitro	Murine fibrosarcoma	Anti-inflammatory	p38, ERK-1/2, C-Jun (JNK)	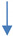	[136]
WA	In vitro	Cellular models of cystic fibrosis inflammation (KKLEB cells)	Anti-inflammatory	NFk-β and IL-8	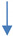	[187]
WA	In vitro	Human melanoma cells (M14, Lu1205, and Sk28)	Anti-cancer	ROS-induced apoptosis increased by lowering the Bax/Bcl2 and Bcl2/Bim ratio	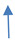	[188]
WA	In vitro	Breast cancer cells (MDA-MB-231 and MCF-7)	Anti-cancer	Caspase-9 and 3 and PARP	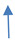	[189]
WA	In vitro, in vivo	Breast tumor progression in xenograft and transgenic mouse models	Anti-cancer	ERK/RSK axis, death receptor 5 (DR-5), ETS domain containing protein-1 (Elk1), and CAAT/enhancer-binding protein-homologous protein (CHOP)	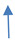	[190]
WA	In vitro	Human laryngeal carcinoma Hep2 cells	Anti-cancer	Cell cycle arrest with concomitant blockade of angiogenesis	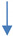	[191]
WA	In vitro	Renal cancers (Caki cells)	Anti-cancer	STAT-3 pathway	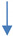	[192]
WA	In vitro	Renal cancers (Caki cells)	Anti-cancer	GRP-78 and CHOP	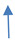	[193]
Extract of *Whitania aristata*	In vivo	Male albino Sprague-Dawley rats and male and female albino Swiss mice	Diuretic effect	Diuretic activity, excretion of sodium and potassium ions	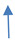	[194]
WA	In vitro	*H. pylori*-induce bone marrow-derived dendritic cells (BMDCs)	Anti-gastric cancer	NF-κB, IL-1β, NLRP3	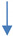	[195]
Aqueous root extract of *Withania somnifera*	In vitro	Nicotine induced conditioned place reference in male albino mice	Anti-addictive	Nicotine efficacy	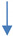	[196]
WA	In vitro	Microglial cells	Anti-inflammatory	STAT1/3, interferon-gamma activated sequence (GAS)-promoter activity	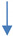	[197]
WA	In vitro	Mouse model of FTLD	Neuroprotective	TAR DNA-binding protein-43, NF-κB activity and neuroinflammation	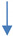	[198]
WA	In vivo and ex vivo	TNF-stimulated human umbilical vein-endothelial cells	Anti-coagulant	Plasminogen activator inhibitor type 1 (PAI-1/t), tissue-type plasminogen activator (t-PA)	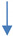	[21]
WA	In vivo	Swiss albino mice	Anti-diabetic	Hyperglycemia		[199]

## 6. Structural Modifications of WA for Further Neuroprotective Activity

**Figure 4:**
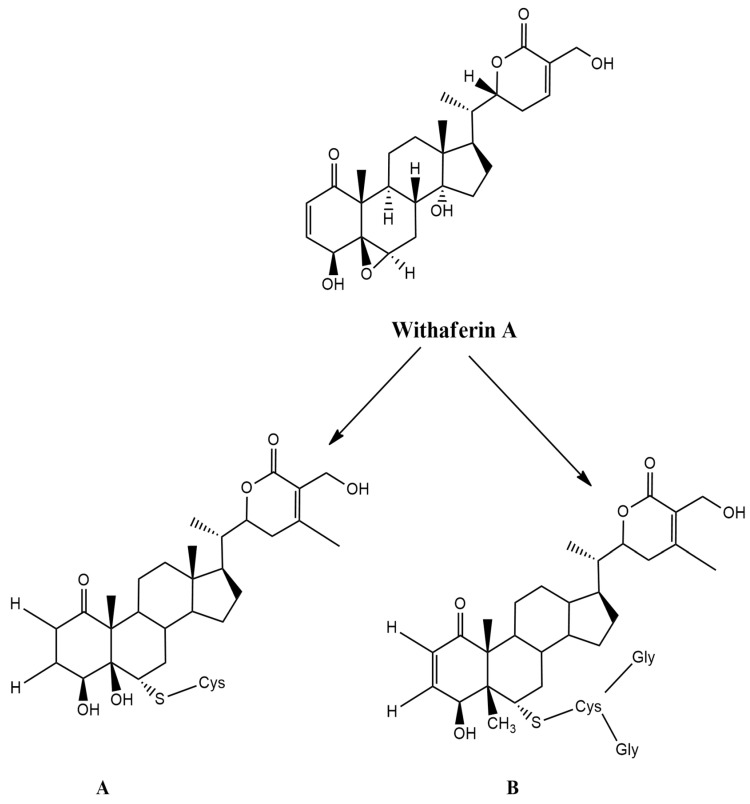
Metabolites of WA: (**A**) cysteine conjugate of WA; (**B**) glutathione conjugate of WA. Here, WA: withaferin A.

One of the methods that could enhance the pharmacological activity of a bioactive compound is through modification of its structure. In this respect, reverse pharmacology approaches have shown that WA has the highest pharmacological potential as a bioactive substance in *W. somnifera* [200]. Evaluation of the WA molecular structure shows three positions that could interact with target proteins. Modification of its structure involves nucleophilic site binding and alkylation reactions at C3 of A-ring at place C3 and the epoxide system at C24 (Figure 4). These sites are most vulnerable to nucleophilic attacks and alkylation reactions.

Published research revealed that the two WA conjugates, glutathione (CR-777) and cysteine (CR-591), exhibit neuroprotective properties in various neurodegenerative disorders. In this respect, WA glutathione (CR-777) conjugate at a nanomolar dose reversed mesencephalic neuron damage caused by alpha-synuclein (α-Syn), 6-hydroxydopamine (6-OHDA), and 1-methyl-4-phenylpyridinium (MPP+). In addition, the WA glutathione conjugate retains neuritis integrity and decreases the overexpression of α-Syn induced by 6-OHDA. These substances activate the PI3K/mTOR pathway, which reduces oxidative stress and inhibits TAU phosphorylation, aggregation of α-Syn, and caspase 3 expression, demonstrating neuroprotective properties [21]. 

## 7. Conclusions and Future Perspectives

Since ancient times, humans have relied on nature as the source of medicine to treat complex diseases, which were managed by primary and secondary metabolites of Ayurvedic, herbal, and medicinal foods. There have been various endeavors to treat AD by reducing Aβ, tau protein level, oxidative stress, and neuroinflammation in the brain. In this context, WA has several specific mechanisms targeting receptors and enzymes in AD. Data collected from this literature review highlight WA as a promising neuroprotective agent to treat AD. It is associated with protein, enzymes, and oxidative and inflammatory markers of AD. Specific markers of AD including amyloid-beta accumulation, tau protein hyperphosphorylation, synaptic changes in the brain, oxidative stress, and inflammatory marker may be treated with WA. Considering the readily available literature pertaining to WA potentiality on AD, future clinical trials should be initiated depending on specific targets. 

Structurally, the transformation of WA in nucleophilic sites and alkylation can be speculated as mechanistic insights in neuro-protection. Although in-depth research on several molecular targets of WA has been identified, the pharmacological efficacy of CNS to treat AD has not been yet discovered in a dose-dependent manner. In addition, even though there is an abundance of in vitro and in vivo studies related to the ability of WA as a potent therapeutic agent to alleviate AD, lack of clinical trials is a critical limitation of WA in AD. In particular, WA exerts anti-Alzheimer activity through various mechanisms of action at cellular and molecular levels. WA exhibits antioxidant or free radical scavenging potential associated with AD. One area that is worth mentioning is the encapsulation of WA in nanoparticles, as means to enhance its delivery into the brain, to improve cognitive disorders; this work is in preclinical and clinical trials. Further mechanistic studies of drug delivery and in vivo efficacy are indispensable parts to analyze the therapeutic role of WA in AD. In summary, we have systematically analyzed the therapeutic efficacy of WA in various animal AD models that furnished unbiased evidence for further execution of WA as a promising therapeutic candidate in AD clinical treatment. WA has actions on multiple targets; however, it can cross the BBB through liposomal nanoformulations, which will be a potent therapeutic candidate to combat AD and AD-like diseases. Since several WA analogs can be important in combatting AD, WA and its analogs is an interesting field of research of therapeutic potential for the maintenance of neuronal health and treatment of AD.
